# Recalibration of the Frontal Behavioural Inventory Cut‐Off for Behavioural Screening in Amyotrophic Lateral Sclerosis

**DOI:** 10.1111/ene.70778

**Published:** 2026-10-07

**Authors:** Barbara Iazzolino, Francesca Palumbo, Antonio Canosa, Stefano Callegaro, Vita Passidomo, Umberto Manera, Rosario Vasta, Enrico Matteoni, Sara Cabras, Giorgio Pellegrino, Alessandra Maccabeo, Cristina Moglia, Andrea Calvo, Adriano Chiò

**Affiliations:** ^1^ ‘Rita Levi Montalcini’ Department of Neuroscience University of Turin Turin Italy; ^2^ Azienda Ospedaliero‐Universitaria Città della Salute e della Scienza di Torino Turin Italy

**Keywords:** amyotrophic lateral sclerosis, behavioural impairment, Frontal Behavioural Inventory, frontotemporal degeneration, psychometric recalibration

## Abstract

**Objective:**

The Frontal Behavioural Inventory (FBI) was validated in behavioural‐variant frontotemporal dementia (bvFTD) with a cut‐off of 25. We tested whether this threshold is appropriate in amyotrophic lateral sclerosis (ALS), where behavioural change is milder and partly confounded by motor pathology.

**Methods:**

We analysed 506 ALS patients with a valid FBI assessment. Behavioural impairment was defined using two complementary reference standards, to minimise dependence on any single behavioural instrument: a consensus rule (pathology on ≥ 2 of three independent instruments: Edinburgh Cognitive and Behavioural ALS Screen, Frontal Systems Behaviour Scale, Beaumont Behavioural Inventory) and Latent Class Analysis. ROC analysis identified the Youden‐optimal cut‐off; agreement was quantified by Cohen's *κ*; internal validity was assessed by stratified 5‐fold cross‐validation. Total and subscale scores were analysed separately.

**Results:**

Among 345 complete‐case patients, 24.6% met criteria for behavioural impairment. The FBI total discriminated impairment well (AUC 0.855, 95% CI 0.806–0.898). The Youden‐optimal cut‐off was FBI ≥ 9 (sensitivity 0.87, specificity 0.74), whereas the legacy cut‐off of 25 showed sensitivity 0.22. Subscale recalibration yielded cut‐offs of FBI‐Apathy ≥ 7 (AUC 0.808) and FBI‐Disinhibition ≥ 4 (AUC 0.851).

**Conclusions:**

In ALS, the bvFTD‐derived threshold of 25 misses most patients with behavioural involvement. FBI ≥ 9 is a promising candidate ALS‐specific screening threshold, with scores ≥ 15 indicating a higher probability of behavioural involvement; exploratory subscale thresholds of ≥ 7 for Apathy and ≥ 4 for Disinhibition are also proposed. These cut‐offs substantially improve sensitivity while maintaining acceptable specificity, but require prospective external validation before replacing current practice.

## Graphical abstract

Using the cut‐off of 25 inherited from frontotemporal dementia, nearly four out of five ALS patients with behavioural impairment go undetected. In 506 patients we recalibrated the Frontal Behavioural Inventory and identified an ALS‐specific screening threshold of ≥ 9, which raised sensitivity from 0.22 to 0.87 while preserving acceptable specificity and remained stable across reference standards and subgroups. FBI ≥ 9 is a promising candidate threshold requiring prospective external validation.

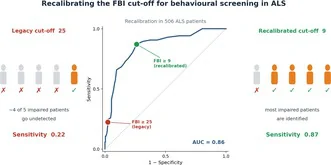

## 1 Introduction

The Frontal Behavioural Inventory (FBI) is widely used to screen for behavioural change in amyotrophic lateral sclerosis (ALS), yet its diagnostic threshold has never been recalibrated for this disease: the cut‐off of 25, applied to ALS from its origins in behavioural‐variant frontotemporal dementia. Applied unchanged to ALS, this threshold misses most behaviourally impaired patients. Cognitive and behavioural change is recognised as an integral feature of ALS and lies on a continuum extending to frontotemporal dementia (FTD), as formalised by the revised Strong criteria [1]. Behavioural changes occur in up to half of patients and are characterised predominantly by apathy, perseveration, irritability and disinhibition [2, 3]. Such changes carry prognostic implications for management decisions, ventilation and gastrostomy [4, 5]. They may be present at diagnosis, often precede overt cognitive impairment, and are associated with shorter survival independently of motor severity [2]. making reliable behavioural screening clinically important [2, 4].

The FBI is a 24‐item caregiver‐rated questionnaire developed by Kertesz et al. for the differential diagnosis of behavioural‐variant FTD (bvFTD) [6]; its Italian version established a total‐score cut‐off of 25 [7], which has been carried into ALS research without formal recalibration. The FBI is routinely used in ALS cohorts across several countries [8, 9, 10, 11, 12, 13]. Applying the legacy threshold unchanged to ALS is problematic for two reasons. First, behavioural change in ALS is generally milder than the florid syndromes of bvFTD, with apathy and irritability predominating and overt disinhibition much less common [3, 14], so a bvFTD‐calibrated threshold is expected to be overly conservative. Second, several FBI items tap motor and bulbar phenomena that may be endorsed in ALS for reasons unrelated to behaviour [15]; ALS‐specific instruments such as the Beaumont Behavioural Inventory were developed explicitly to address these confounds [16].

Systematic reviews of behavioural screening in ALS have noted that cut‐off scores for most instruments, including the FBI, derive from non‐ALS populations [15]; a further review of validated screening methods for ALS [17] likewise identified no validated FBI cut‐off for non‐demented ALS patients.

The clinical cost of this omission is substantial: as we show below, at the legacy cut‐off of 25 nearly four out of five behaviourally impaired ALS patients remain undetected. Therefore, the aims of this study were to: (i) determine the optimal FBI cut‐off for behavioural impairment in a large ALS cohort, (ii) assess the robustness and internal validity of that cut‐off and (iii) quantify the improvement in agreement relative to the legacy cut‐off of 25.

## 2 Materials and Methods

### 2.1 Participants

We retrospectively analysed 506 patients with ALS according to the El Escorial revised [18] or Gold Coast criteria [19], consecutively assessed at the Turin CRESLA Centre between 2018 and 2025, each contributing a single baseline FBI assessment. Of these, 345 with concurrent ECAS‐behavioural, FrSBe, and BBI assessments constituted the complete‐case subset used for the primary recalibration; the full cohort of 506 was used for the score distribution and pairwise sensitivity analyses.

### 2.2 Instruments

The FBI comprises 24 caregiver‐rated items (0–3 each; total 0–72), grouped into apathy (items 1–12) and disinhibition (items 13–24) clusters [7]; total and subscale scores were analysed separately. We used the original 24‐item FBI rather than the ALS‐modified version (FBI‐ALS) [20], since the original remains the most widely used form in ALS behavioural research and is the version for which the legacy cut‐off of 25 was derived.

Behavioural impairment was independently indexed with three instruments: the behavioural domain of the Edinburgh Cognitive and Behavioural ALS Screen (ECAS), using the Italian pathological threshold [21]; the Frontal Systems Behaviour Scale (FrSBe) [22]; and the Beaumont Behavioural Inventory (BBI), an ALS‐specific caregiver questionnaire designed to capture frontotemporal‐spectrum behavioural change while accounting for motor disability [16]. FBI subscales were additionally anchored to the corresponding FrSBe subscales as a construct‐specific sensitivity analysis.

### 2.3 Reference Standards

No single instrument provides an unambiguous criterion for behavioural impairment in ALS, and any single‐instrument standard would risk circularity with the FBI. We therefore used two complementary reference standards: a consensus rule (pathology on at least two of ECAS‐behavioural, FrSBe total, and BBI) and a model‐based Latent Class Analysis (LCA), fitted both as a three‐indicator model (LCA‐3) and an extended seven‐indicator model (LCA‐7). A two‐class impaired/non‐impaired solution was retained throughout to preserve a clinically interpretable binary framework. The full LCA specification, including estimation, class selection, and the bootstrap likelihood‐ratio test, is given in Appendix S1.

### 2.4 Statistical Analysis

FBI discrimination of each reference standard was quantified by the area under the ROC curve (AUC), and the Youden‐optimal integer cut‐off was identified; a cost‐ratio analysis, weighting false negatives more heavily than false positives to reflect the greater cost of missed impairment in screening, confirmed its appropriateness. Performance against the legacy cut‐off of 25 was characterised by sensitivity, specificity, predictive values, and Cohen's *κ*, interpreted by the Landis–Koch convention [23].

Calibration (a quadratic logistic model with Spiegelhalter and Cox tests) and clinical utility (Decision Curve Analysis) were assessed as detailed in Appendix S1. Internal validity used 2000‐resample bootstrap confidence intervals for the AUC and cut‐off, a stratified 70/30 train–test split, 5‐fold cross‐validation, and the 0.632+ bootstrap for optimism correction.

### 2.5 Additional Sensitivity Analyses

Three analyses addressed motor confounding: ROC analysis and cut‐off identification repeated after stratifying by ALSFRS‐R severity; Spearman correlations between FBI scores and the four ALSFRS‐R functional domains; and the distribution of FBI scores across three decision bands, quantified with Wilson 95% confidence intervals.

Detailed numerical results from all additional analyses, together with the full calibration and decision‐curve methods, are reported in (Appendix S1: Tables S1–S5 and Figure S1). All analyses were performed in Python 3.12 and the code is available at [24].

### 2.6 Standard Protocol Approvals, Registrations, and Patient Consents

The study was approved by the Ethics Committee of the ALS Expert Center of Torino (Comitato Etico Azienda Ospedaliero‐Universitaria Città della Salute e della Scienza, Torino, #0036344, #0038876 and #0064510). Patients provided written informed consent before enrollment. The database was anonymized according to Italian law for the protection of privacy.

## 3 Results

Among the 506 ALS patients with a valid FBI assessment (mean age at testing 66.0 ± 11.2 years; 56.1% male; 29.6% bulbar onset where recorded), the FBI total score was right‐skewed (median 6, mean 9.1 ± 9.0, range 0–72), consistent with predominantly mild behavioural change in most patients and marked impairment in a minority (Table 1). In the 345‐patient complete‐case subset, the consensus reference standard classified 24.6% of patients as behaviourally impaired (85 of 345).

**TABLE 1:** Demographic and clinical characteristics of the cohort.

Characteristic	FBI cohort (*n* = 506)	Complete‐case (*n* = 345)	*p* ^a^
Age at test, years (mean ± SD)	66.0 ± 11.2	65.5 ± 11.1	0.089
Male sex, *n* (%)	284 (56.1%)	198 (57.4%)	—
Education, years (mean ± SD)	10.7 ± 4.2	10.8 ± 4.1	0.264
Site of onset (*n* with data)	506	345	0.067
Bulbar, *n* (%)	150 (29.6%)	93 (27.0%)	
Spinal, *n* (%)	356 (70.4%)	252 (73.0%)	
ALSFRS‐R total at assessment (mean ± SD)	38.7 ± 5.2 (*n* = 411)	39.0 ± 5.1 (*n* = 272)	0.095
MiToS stage (*n* with data)	382	246	0.992
MiToS stage 0/1/2, n	322/57/3	207/37/2	
King's stage (*n* with data)	382	246	0.266
King's Stage 1/2/3/4, *n*	136/122/112/12	94/71/72/9	
*C9orf72* expansion (positive/tested)	25/475 (5.3%)	18/323 (5.6%)	—
Strong 2017 classification (*n* with data)	506	345	0.440
Strong 2017 classification, *n*			
Cognitively normal	218	151	
ALSci	107	74	
ALSbi	79	52	
ALScbi	77	55	
ALS‐FTD	25	13	
FBI total (mean ± SD; median, range)	9.1 ± 9.0; 6 (0–72)	9.4 ± 9.2; 7 (0–72)	0.308
Behavioural impairment by consensus, *n* (%)	—	85 (24.6%)	—

*Note:* Continuous variables: mean ± SD; categorical variables: *n* (%).

Abbreviations: ALSFRS‐R, Amyotrophic Lateral Sclerosis Functional Rating Scale‐Revised; Complete‐case, patients with concurrent ECAS‐behavioural, FrSBe and BBI data; SD, standard deviation; —, not applicable.

^a^ *p*‐value compares complete‐case (*n* = 345) vs. non‐complete‐case (*n* = 161) patients: Mann–Whitney *U* for continuous variables; chi‐square or Fisher exact for categorical variables. *p* shown only for the summary row of grouped variables (e.g., onset site, MiToS, King's, Strong 2017); subcategory rows left blank to avoid redundancy.

The 345 complete‐case patients did not differ significantly from the 161 excluded for incomplete reference‐standard data on any available demographic, clinical, or genetic characteristic, nor in FBI score or the prevalence of behavioural involvement (all *p* > 0.05; Table S1), arguing against material selection bias.

### 3.1 Main Result

#### 3.1.1 ROC Analysis and Optimal Cut‐Off

The FBI total score discriminated the consensus reference standard well (AUC 0.855, 95% CI 0.806–0.898; Figure 1). The Youden‐optimal cut‐off was FBI ≥ 9 (sensitivity 0.87, specificity 0.74; bootstrap CI 8 to 15). At the cohort prevalence of 24.6%, this gave a positive predictive value of 0.52 and a negative predictive value of 0.95, values that will vary with the underlying prevalence. By contrast, the legacy cut‐off of 25 yielded a sensitivity of only 0.22, missing approximately four out of five patients classified as impaired by the reference standard (Table 2). Increasing the cut‐off above 9 progressively traded sensitivity for specificity: in an exploratory, post hoc analysis, FBI ≥ 15 retained 66% sensitivity at 90% specificity and maximised agreement (Cohen's *κ*), and we describe it below as a higher‐specificity threshold rather than a diagnostic confirmation. When false negatives were weighted three times more than false positives, the cost‐minimising threshold also converged to FBI ≥ 9, supporting the clinical appropriateness of the Youden‐derived threshold.

**FIGURE 1:**
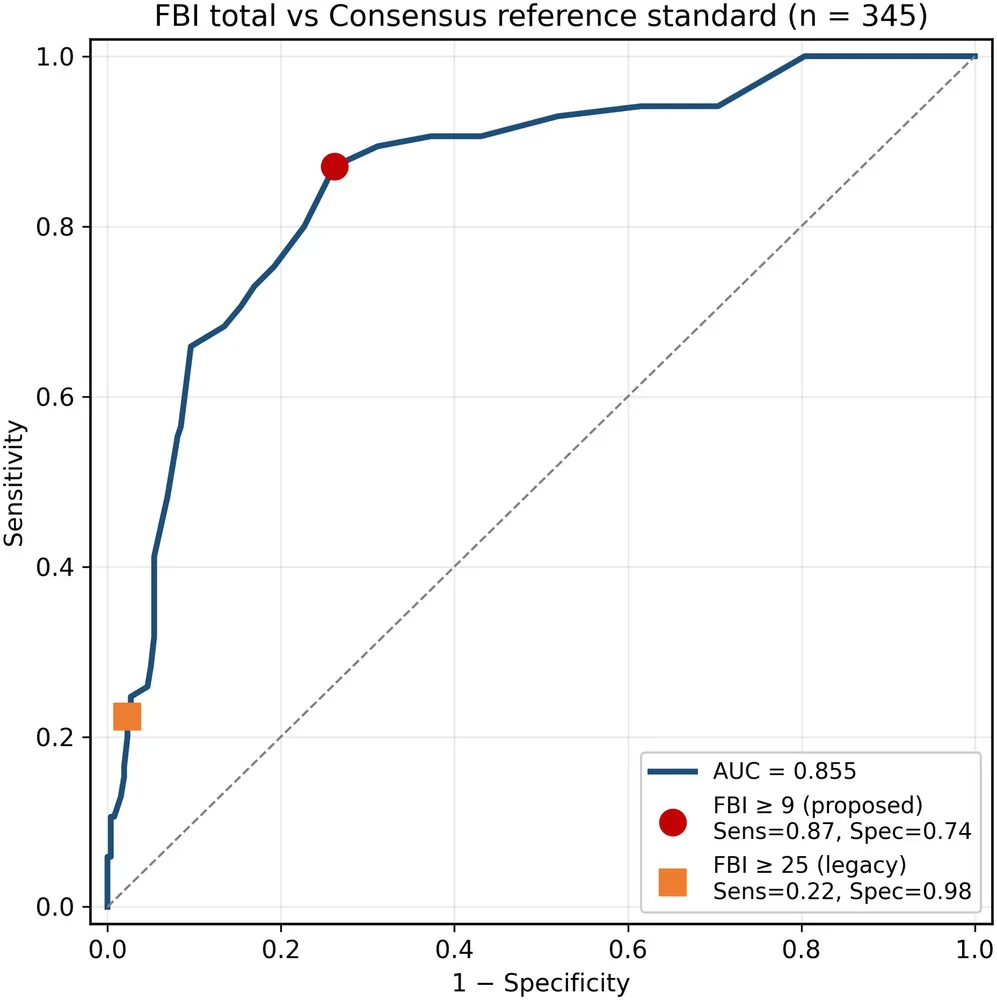
Receiver operating characteristic curve for the FBI total score against the consensus behavioural reference standard. The curve displays overall discrimination performance, with markers indicating the operating points corresponding to the recalibrated cut‐off and the legacy threshold.

**TABLE 2:** Performance of candidate FBI total cut‐offs against the consensus reference standard.

Cut‐off	Sensitivity	Specificity	PPV	NPV	Cohen's *κ*
FBI ≥ 8	0.894	0.688	0.484	0.952	+0.453
FBI ≥ 9	0.871	0.738	0.521	0.946	+0.497
FBI ≥ 10	0.800	0.773	0.535	0.922	+0.491
FBI ≥ 12	0.729	0.831	0.585	0.904	+0.517
FBI ≥ 15	0.659	0.904	0.691	0.890	+0.572
FBI ≥ 25 (legacy)	0.224	0.977	0.760	0.794	+0.263

*Note:* Performance of candidate FBI total cut‐offs against the consensus reference standard (*n* = 345, behavioural‐impairment prevalence 24.6%). The proposed ALS cut‐off is Youden‐optimal; FBI ≥ 25 is the legacy bvFTD‐derived threshold, shown for comparison.

Abbreviations: NPV, negative predictive value; PPV, positive predictive value.

#### 3.1.2 Agreement With the Reference Standards

Cohen's *κ* between the dichotomized FBI total and each reference standard improved substantially with the new cut‐off relative to the legacy threshold, against every reference standard examined (Table 4). The gain ranged from +0.15 against the single‐instrument FrSBe standard to +0.40 against the BBI, +0.41 against LCA‐3, and +0.38 against LCA‐7, with +0.23 against the consensus rule. These improvements moved the dichotomised FBI from negligible‐to‐weak agreement (*κ* 0.14–0.26) at the legacy cut‐off to moderate‐to‐substantial agreement (*κ* 0.33–0.57) at the new cut‐off, consistent with the Landis–Koch convention [23].

### 3.2 Robustness Analyses

A central feature of the recalibration is that the optimal cut‐off fell within a narrow range across analyses, from 8 to 10 against the different reference standards and clinically relevant subgroups (Table 4). For a screening instrument, where failing to detect impairment is more costly than a false alarm, we adopt the lower, screening‐appropriate bound, FBI ≥ 9. The following analyses were designed less to find the cut‐off than to test whether this value could be an artefact of the reference standard, of motor disability, or of the internal validation strategy.

#### 3.2.1 Robustness Across Reference Standards

Both LCA models yielded a two‐class impaired/non‐impaired solution (BLRT *p* < 0.001 in each), retained throughout because the aim was a binary external reference standard rather than phenotype discovery; the latent impaired class comprised 42.6% of patients in LCA‐3 and 31.0% in LCA‐7 (full model diagnostics in Appendix S1). The Youden‐optimal FBI cut‐off was 8 against LCA‐3 (AUC 0.834) and 9 against LCA‐7 (AUC 0.821), consistent with the cut‐off of 9 identified against the consensus standard.

#### 3.2.2 Internal Validation

A stratified 70/30 train–test split returned a training cut‐off of FBI ≥ 9 that retained sensitivity 0.96 and specificity 0.71 in the held‐out set; 5‐fold cross‐validation returned FBI ≥ 9 in all five folds (mean test sensitivity 0.87, specificity 0.74). The 0.632+ bootstrap gave an optimism‐corrected AUC of 0.855 and misclassification rate of 0.229, numerically identical to the apparent estimates, confirming negligible optimism bias.

#### 3.2.3 Psychometric Properties

Item‐level FBI scores were available for 294 complete‐case patients. Internal consistency was high (Cronbach's *α* 0.89), sampling adequacy was satisfactory, and exploratory factor analysis showed a dominant general factor consistent with use of the total score, alongside an interpretable apathy/disinhibition structure (Table S2). The aphasia and alien‐hand items loaded weakly on all factors, in keeping with their limited behavioural specificity in ALS.

#### 3.2.4 Convergent and Divergent Validity

The FBI total correlated strongly with the other behavioural instruments (BBI and FrSBe), and its subscales with the matched FrSBe subscales, whereas correlations with the HADS anxiety and depression subscales were weak and non‐significant (Table S3), indicating that the FBI captures behavioural change rather than emotional distress.

#### 3.2.5 Item‐Response Analysis

A two‐parameter item‐response model showed good discrimination for most items, with difficulty ordered coherently for ALS (Table S2, Figure S2). The aphasia and alien‐hand items again had the lowest discrimination, consistent with their susceptibility to motor confounding.

#### 3.2.6 Independent Expert Reference Standard

Because the consensus and latent‐class standards are both derived from caregiver questionnaires, we repeated the analysis against an independent expert standard, the clinical Strong ALS‐FTSD classification assigned by the treating clinicians. Median FBI scores rose monotonically across classes (Table S9), and against this standard the FBI total discriminated behavioural involvement well (AUC 0.83), with a Youden‐optimal cut‐off of 10 (bootstrap CI 6 to 12), close to the consensus‐derived value; the legacy cut‐off of 25 again showed very low sensitivity.

#### 3.2.7 Calibration

The estimated probability of behavioural involvement rose monotonically with FBI score, crossing 20% at the new cut‐off, about 43% at FBI ≥ 15, and exceeding 75% at the legacy cut‐off (Figure 2). Because this model was fitted and evaluated in the same cohort, it describes the internal coherence of predicted probabilities rather than providing independent validation of the cut‐off; on this basis, no systematic miscalibration was evident (Figure 2; Appendix S1). Moving from the legacy cut‐off of 25 to FBI ≥ 9 captured an additional 65 percentage points of patients classified as impaired by the reference standard (22%–87%), at a cost of 24 percentage points of specificity.

**FIGURE 2:**
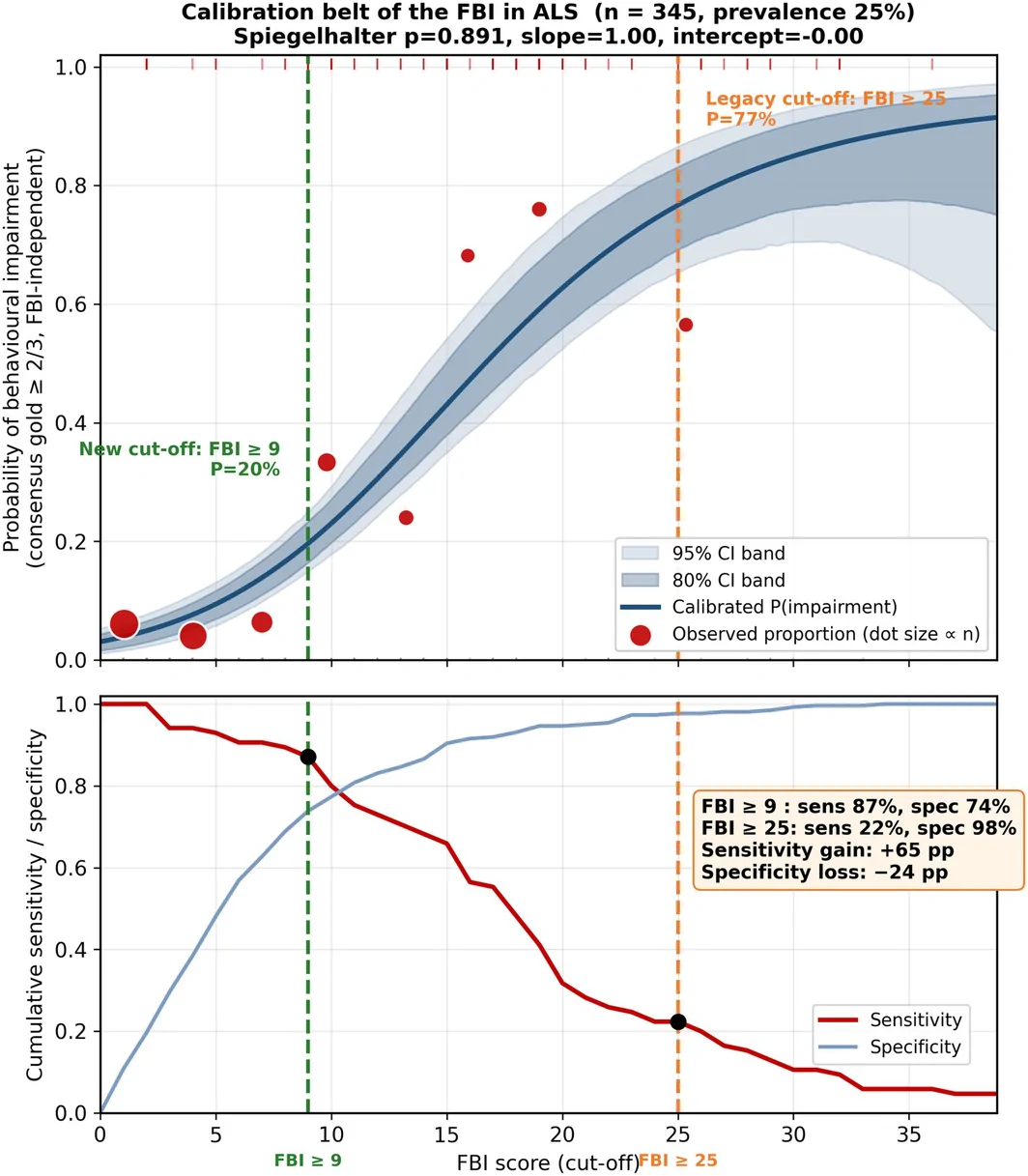
Calibration belt for the quadratic logistic model relating FBI total score to the probability of behavioural impairment. The plot shows model‐predicted probabilities with bootstrap confidence bands, observed impairment proportions across score bins, and the positions of the recalibrated and legacy cut‐offs.

#### 3.2.8 Subscale Recalibration

The FBI Apathy and Disinhibition subscales each discriminated their construct‐matched reference well, giving exploratory Youden‐optimal cut‐offs of FBI‐Apathy ≥ 7 and FBI‐Disinhibition ≥ 4 (Table 3). These are secondary analyses against single‐instrument anchors rather than the multi‐instrument standard used for the total score, and the disinhibition threshold rests on the low base rate of florid disinhibition in ALS; they therefore require independent validation before clinical use. These subscale thresholds are exploratory: they were derived as secondary analyses against single‐instrument, construct‐matched anchors rather than the multi‐instrument standard used for the total score, and the disinhibition threshold in particular rests on the low base rate of florid disinhibition in ALS. They therefore require independent validation before clinical use.

**TABLE 3:** Subscale recalibration.

Subscale	Anchor	*n*	AUC (95% CI)	Optimal cut‐off (95% CI)	Sens./Spec.	Cohen's *κ*
FBI apathy	Consensus ≥ 2/3	345	0.808 (0.755–0.861)	≥ 7 (5–11)	0.80/0.72	+0.430
FBI apathy	FrSBe‐apathy	345	0.815 (0.769–0.862)	≥ 8 (6–9)	0.77/0.78	+0.491
FBI disinhibition	Consensus ≥ 2/3	345	0.851 (0.804–0.894)	≥ 4 (2–5)	0.65/0.88	+0.526
FBI disinhibition	FrSBe‐disinhibition	345	0.809 (0.745–0.872)	≥ 2 (2–8)	0.94/0.58	+0.202
FBI disinhibition	ECAS‐disinhibition	345	0.776 (0.668–0.866)	≥ 4 (2–7)	0.67/0.78	+0.182

*Note:* Subscale recalibration. Youden‐optimal cut‐offs for the FBI Apathy/negative (items 1–12) and Disinhibition (items 13–24) subscales, against the multi‐instrument consensus reference standard and against construct‐matched ECAS/FrSBe subscales. Bootstrap 95% CI from 2000 resamples.

Abbreviation: AUC, area under the ROC curve.

### 3.3 Analyses Addressing Motor Confounding

The central threat to a behavioural score in ALS is that it may reflect physical disability rather than behaviour: a patient may appear apathetic because of anarthria or immobility, and the FBI total may rise because motor function declines. The following three analyses were designed to assess the potential influence of motor disability.

#### 3.3.1 Sensitivity Analysis by Site of Onset

Recalibration repeated separately in bulbar and spinal onset showed no significant difference in FBI scores between groups and a non‐significant FBI‐by‐onset‐site interaction, indicating that the FBI–impairment relationship does not differ by onset site. Discrimination was excellent in both subgroups and FBI ≥ 9 performed well in each, whereas the legacy cut‐off of 25 showed very low sensitivity in both. A single cut‐off of 9 therefore appears appropriate across onset sites (Table S4) showed very low sensitivity in both (0.30 and 0.19). A single cut‐off of 9 therefore appears appropriate across onset sites (Table S4).

#### 3.3.2 Sensitivity by Motor Severity

Stratifying by ALSFRS‐R severity, patients with greater disability showed higher FBI scores (median 10 vs. 5), but discrimination remained excellent in both strata, the Youden‐optimal cut‐off was FBI ≥ 9 in each, and the FBI‐by‐severity interaction was non‐significant, indicating that the FBI–impairment relationship does not differ by motor severity (Table S5).

#### 3.3.3 Construct Validity vs. Motor Disability

Spearman correlations between FBI scores and the ALSFRS‐R, including its four functional domains, were modest and all well below the |*ρ*| ≥ 0.30 threshold for substantial method overlap (Table S6), indicating that motor disability has at most a modest influence on FBI scoring at the level of the total score. Consistent with this, agreement was highest against the BBI (Δ*κ* +0.40; Table 4), the reference instrument explicitly designed to capture behavioural change while accounting for motor disability.

**TABLE 4:** Cohen's *κ* of the dichotomised FBI total against six reference standards, at the proposed (≥ 9) and legacy (≥ 25) cut‐offs.

Reference standard	*n*	Sens. at ≥ 9	*κ* at ≥ 9	*κ* at ≥ 25	Δ*κ*
ECAS‐behavioural	345	0.750	+0.331	+0.167	+0.164
FrSBe total	345	0.857	+0.367	+0.214	+0.153
BBI	345	0.736	+0.558	+0.156	+0.402
Consensus ≥ 2/3	345	0.871	+0.497	+0.263	+0.234
LCA‐3	345	0.735	+0.565	+0.151	+0.415
LCA‐7	345	0.804	+0.521	+0.142	+0.380

*Note:* Cohen's *κ* of the dichotomised FBI total against six reference standards, at the proposed (≥ 9) and legacy (≥ 25) cut‐offs. Δ*κ* = improvement with the new cut‐off. *n* = 345 for all comparisons.

### 3.4 Clinical Translation

#### 3.4.1 Distribution Across Decision Bands

FBI scores showed a stable three‐band distribution: most patients scored below the screening cut‐off, with smaller proportions in the intermediate 9–14 band and at or above 15, and the prevalence of consensus‐defined behavioural impairment increased monotonically across the three bands (Table S7).

Decision Curve Analysis (Figure S1) showed that FBI ≥ 9 provided the greatest net benefit across the screening range (threshold probability ≲ 0.30), while FBI ≥ 15 gave greater benefit at higher probabilities and the legacy ≥ 25 rule was inferior throughout, supporting the two‐threshold structure (Table S8). At a threshold probability of 0.25, FBI ≥ 9 avoided about 46 unnecessary interventions per 100 patients versus ≥ 25. The continuous model, fitted and evaluated in the same cohort, is optimistic relative to the fixed cut‐offs.

## 4 Discussion

The central finding of this study is that the optimal FBI cut‐off for behavioural screening in ALS falls well below the bvFTD‐derived threshold of 25, within a narrow range around 9, consistently across reference‐standard definitions and across subgroups defined by site of onset and disease severity. In a cohort of 506 ALS patients, this threshold raised sensitivity from 0.22 to 0.87 while preserving acceptable specificity (0.74), supporting replacement of the legacy cut‐off. We acknowledge one conceptual limitation at the outset: no behavioural reference standard in ALS is fully independent of the construct measured by the FBI, which is why we combined several instruments with latent class analysis rather than relying on any single one. That conceptually distinct approaches and clinically distinct subgroups all pointed to the same narrow range argues against the result being an artefact of any single analytical choice. The improvement was driven by better identification of patients classified as impaired by the reference standard rather than a shift in marginal frequencies, with Cohen's *κ* rising from negligible‐to‐weak at the legacy cut‐off to moderate‐to‐substantial at the new threshold.

The lower cut‐off required in ALS reflects the milder behavioural phenotype, with florid disinhibition uncommon and several items capturing motor or bulbar rather than behavioural phenomena; together these shift the FBI distribution toward lower values [3, 14, 15].

Our approach differs from that of Consonni et al. [25], who excluded five putatively confounded items a priori and analysed the remaining scale dimensionally without proposing an operational threshold. We instead preserved the original 24‐item scale and recalibrated its threshold statistically against multiple reference standards, providing a clinically usable cut‐off while assessing potential motor confounds through dedicated sensitivity analyses.

Several other behavioural instruments are validated in Italian ALS populations, and the choice among them depends on the aim: the ECAS behavioural domain and the ALS‐CBS behavioural questionnaire are brief clinical screens, while the Beaumont Behavioural Inventory, designed to minimise motor confounding, is preferable when motor disability is advanced. The recalibrated FBI is most useful where continuity with the extensive FBI‐based literature is desired, or where it is already embedded in a centre's battery.

The clinical implications are direct: behavioural screening in ALS informs decision‐making capacity, care planning, trial eligibility, and prognostic communication [4, 5], where a threshold that misses most impaired patients has direct consequences. The proposed cut‐off of ≥ 9 closes most of that gap, raising the proportion of behaviourally impaired patients correctly identified from 22% to 87%. The exploratory subscale cut‐offs may add phenotypic information, the apathy subscale capturing the dominant dimension in ALS. We also explored, post hoc, a higher threshold for use where specificity is prioritised: FBI ≥ 15 offers tighter specificity (Table 2) and may suit selection of research cohorts, but because roughly a third of patients scoring ≥ 15 did not meet the reference standard, we describe it as a higher‐specificity threshold, not a confirmatory one. This two‐threshold structure was not pre‐specified and is exploratory, and is broadly compatible with cut‐off ranges reported for ALS‐specific instruments such as the BBI, though direct comparison should be cautious [16].

Across three complementary analyses, site of onset, disease severity, and correlations with the ALSFRS‐R, we found that motor disability had at most a limited influence on the relationship between FBI scores and behavioural impairment: FBI ≥ 9 performed comparably in bulbar and spinal onset and in mild and moderate‐to‐severe disease; formal FBI‐by‐subgroup interaction tests were non‐significant, and correlations between FBI scores and every ALSFRS‐R motor domain were weak (all |*ρ*| ≤ 0.21).

Robustness was further supported by internal validation, with bootstrap and cross‐validation confirming negligible optimism bias.

Decision‐curve analysis supported the same two‐threshold interpretation, with FBI ≥ 9 giving the greatest net benefit in the screening range and the ≥ 15 cut‐off approaching the continuous model at higher threshold probabilities. In this context, a positive FBI screen does not trigger a specific treatment but referral for structured behavioural and neuropsychological assessment and, where relevant, adjustment of care planning and caregiver support. Because this downstream action carries little risk while missing behavioural involvement is costly, the low‐to‐moderate threshold‐probability range we examined is the clinically plausible one for such a screen. As with any screening instrument, a positive result identifies patients for whom structured neuropsychological evaluation is warranted, consistent with the expert‐judgement basis of the Strong ALSbi criteria [1].

The distribution of FBI scores also revealed a clinically interpretable intermediate range (9–14), characterised by an impairment prevalence that was neither negligible nor decisively high. This grey zone likely captures patients with subtle or emerging changes who may benefit from monitoring rather than immediate categorical classification.

Limitations include the single‐centre design, the absence of an external validation cohort, and reliance on instrument‐based rather than expert‐consensus criteria for the reference standard. A further limitation concerns the instrument itself: because we used the original FBI, several items, notably aphasia/verbal apraxia and alien‐hand/apraxia, may be endorsed for motor or bulbar rather than behavioural reasons, and our item‐level analyses confirmed that these items had the weakest discrimination (Table S2, Figure S2); an ALS‐adapted FBI might further sharpen the threshold. Because any reference standard built from overlapping caregiver instruments carries some risk of shared method variance, the latent‐class prevalence estimates in particular should be interpreted with caution. The convergence of cut‐off estimates across the consensus rule and latent class analysis partially mitigates but does not eliminate this concern, and the recalibrated cut‐off should be confirmed prospectively in an independent, ideally multicentre ALS cohort incorporating expert Strong classification.

Future work should test the recalibrated cut‐off in independent ALS cohorts, particularly non‐Italian populations, where ALS‐specific instruments such as the BBI may outperform the FBI [24], and in longitudinal designs [11], where the FBI could be used to track behavioural progression within the 9–14 grey zone and inform the timing of supportive interventions.

These findings also illustrate a broader methodological principle: neuropsychological instruments validated in one disease cannot simply be transferred to another while retaining the same diagnostic threshold because both the distribution of the underlying construct and the behaviour of individual items change across diseases. The FBI in ALS provides an instructive example of this principle. In conclusion, the present findings support recalibration of the FBI for ALS, with a cut‐off of ≥ 9 providing substantially better sensitivity than the traditional threshold of 25, while maintaining acceptable specificity. Pending replication in independent cohorts, this threshold may improve identification of clinically meaningful behavioural change in ALS patients across clinical and research settings.

## Author Contributions

**Barbara Iazzolino:** conceptualization, investigation, writing – review and editing, supervision, writing – original draft, data curation. **Francesca Palumbo:** conceptualization, investigation, writing – original draft, writing – review and editing. **Antonio Canosa:** conceptualization, writing – review and editing. **Stefano Callegaro:** conceptualization, formal analysis, data curation, software. **Vita Passidomo:** conceptualization, investigation, writing – review and editing. **Umberto Manera:** conceptualization, writing – review and editing, project administration. **Rosario Vasta:** conceptualization, writing – review and editing, methodology, investigation. **Enrico Matteoni:** conceptualization, writing – review and editing. **Sara Cabras:** conceptualization, investigation, writing – review and editing. **Giorgio Pellegrino:** conceptualization, investigation, writing – review and editing. **Alessandra Maccabeo:** conceptualization, writing – review and editing. **Cristina Moglia:** conceptualization, investigation, writing – review and editing, visualization. **Andrea Calvo:** conceptualization, writing – review and editing, writing – original draft, validation. **Adriano Chiò:** funding acquisition, conceptualization, writing – original draft, writing – review and editing, data curation, supervision, resources, validation.

## Funding

This work was supported by the Italian Ministry of Health (Ministero della Salute, Ricerca Sanitaria Finalizzata, grant RF‐2016‐02362405); the Progetti di Rilevante Interesse Nazionale program of the Ministry of Education, University and Research (grants 2017SNW5MB and 20228N7573); the European Commission's Health Seventh Framework Programme (project EUROMOTOR under grant agreement 259867); the Horizon 2020 Programme (project Brainteaser under grant agreement 101017598); the Horizon Europe Programme (project Hereditary under grant agreement 101137074); the Joint Programme–Neurodegenerative Disease Research (Strength, ALS‐Care and Brain‐Mend projects), granted by the Italian Ministry of Education, University and Research; and Agenzia per la Ricerca sulla SLA (ARISLA) (project DIG‐ALS). This study was performed under the Department of Excellence grant of the Italian Ministry of University and Research to the “Rita Levi Montalcini” Department of Neuroscience, University of Torino, Italy.

## Ethics Statement

The study was conducted in accordance with the Declaration of Helsinki and was approved by the Ethics Committee of the ALS Expert Center of Torino (Comitato Etico Azienda Ospedaliero‐Universitaria Città della Salute e della Scienza, Torino, #0036344, #0038876 and #0064510).

## Consent

All participants provided written informed consent before enrollment. The database was anonymized according to Italian law for the protection of privacy.

## Conflicts of Interest

The authors declare no conflicts of interest.

## Supporting information


**Table S1:** Comparison of complete‐case and excluded patients.
**Table S2:** Item‐level properties of the Frontal Behavioural Inventory (*n* = 294).
**Table S3:** Convergent and divergent validity of the Frontal Behavioural Inventory.
**Table S4:** Sensitivity analysis—bulbar vs spinal onset.
**Table S5:** Sensitivity analysis by Motor Severity (ALSFRS‐R).
**Table S6:** Spearman correlations between FBI scores and ALSFRS‐R domain scores.
**Table S7:** Distribution of FBI scores across decision bands.
**Table S8:** Net benefit at representative threshold probabilities (Decision Curve Analysis).
**Table S9:** FBI total score and diagnostic performance against the independent expert clinical (Strong ALS‐FTSD) classification.
**Figure S1:** Decision Curve Analysis of FBI cut‐offs in ALS (*n* = 345). Net benefit is plotted against the threshold probability of behavioural impairment (p_t) for five strategies: FBI continuous (logistic model), FBI ≥ 9 (proposed screening cut‐off), FBI ≥ 15 (exploratory higher‐specificity cut‐off), FBI ≥ 25 (legacy cut‐off), and the treat‐all and treat‐none baselines. The shaded band (p_t 0.10–0.40) marks the clinically relevant range for behavioural screening in ALS. The vertical line marks the empirical prevalence (24.6%). Across the entire clinical range, FBI ≥ 9 and the continuous FBI model dominate the treat‐all, treat‐none and legacy ≥ 25 strategies; FBI ≥ 9 is preferable in the screening‐oriented sub‐range (p_t ≲ 0.30), while FBI ≥ 15 gives greater net benefit at higher threshold probabilities, supporting the exploratory two‐threshold structure.
**Figure S2:** Item difficulty and discrimination of the Frontal Behavioural Inventory from a two‐parameter item‐response model (*n* = 294). Items are ordered by difficulty (b): negative/apathy items (blue) are generally endorsed at lower thresholds than disinhibition items (orange), consistent with the milder, apathy‐predominant behavioural phenotype of ALS. Marker size is proportional to discrimination (a). The two motor or bulbar loaded items, aphasia/verbal apraxia and alien‐hand/apraxia (red outline), show the lowest discrimination.

## Data Availability

The PARALS clinical data are available from the corresponding author upon reasonable request, subject to ethical approval. Analysis code reproducing the results is available at https://github.com/Tety94/als‐fbi.
