# Supplementary material for: Recalibration of the Frontal Behavioural Inventory Cut‐Off for Behavioural Screening in Amyotrophic Lateral Sclerosis
Source: Eur J Neurol. 2026 Oct 7;33(10):e70778. doi: 10.1111/ene.70778 (PMC13643373; doi:10.1111/ene.70778)
Supplement: Supplementary file 1 [file ENE-33-e70778-s001.docx]

**Supplementary Statistical Methods**

**Calibration.** The relationship between FBI score and the probability of behavioural impairment was modelled with a quadratic logistic model, logit(p) = β0 + β1·FBI + β2·FBI², fitted across the full FBI range and visualised as a calibration belt with bootstrap confidence bands. Overall miscalibration was tested with Spiegelhalter’s z-test, and calibration slope and intercept were estimated by Cox calibration regression, both evaluated at the observed FBI scores.

**Decision Curve Analysis.** Clinical utility was assessed following Vickers and Elkin (2006). Net benefit was computed across a threshold-probability grid from 0.01 to 0.60 (200 equally spaced points) as NB(pt) = TP/N − (FP/N) × pt/(1−pt), where pt is the clinician’s threshold probability of behavioural impairment above which intervention is warranted, for the strategies FBI ≥ 9, FBI ≥ 12, FBI ≥ 25, a continuous quadratic logistic model of impairment probability as a function of FBI score, and the treat-all and treat-none baselines. The analysis was restricted to the complete-case subset (n = 345). The continuous logistic model was estimated and evaluated on the same sample; its net-benefit estimates are therefore optimistic relative to the fixed cut-off strategies and should be interpreted accordingly.

**Bootstrap and internal validation.** Bootstrap 95% confidence intervals for the AUC and the cut-off were obtained from 2000 stratified percentile resamples, positives and negatives resampled separately to preserve the class ratio. Internal validity was assessed by a stratified 70/30 train–test split and stratified 5-fold cross-validation, with the Youden criterion re-applied in full within each training partition; the .632+ bootstrap (Efron and Tibshirani, 1997) estimated optimism in both AUC and misclassification rate at the chosen cut-off.

**Supplementary References**

Cox, D. R. (1958). Two further applications of a model for binary regression. Biometrika, 45(3–4), 562–565.

Efron, B., & Tibshirani, R. (1997). Improvements on cross-validation: the .632+ bootstrap method. Journal of the American Statistical Association, 92(438), 548–560.

Spiegelhalter, D. J. (1986). Probabilistic prediction in patient management and clinical trials. Statistics in Medicine, 5(5), 421–433. https://doi.org/10.1002/sim.4780050506

Vickers, A. J., & Elkin, E. B. (2006). Decision curve analysis: a novel method for evaluating prediction models. Medical Decision Making, 26(6), 565–574. <https://doi.org/10.1177/0272989X06295361>

**Table S1. Comparison of complete-case and excluded patients.**

| **Characteristic** | **Complete-case (n = 345)** | **Excluded (n = 161)** | **p** |
| --- | --- | --- | --- |
| Age at testing, years, mean (SD) | 65.5 (11.1) | 67.2 (11.4) | 0.089 |
| ALSFRS-R total, mean (SD) | 39.0 (5.1) | 38.2 (5.2) | 0.095 |
| FBI total score, mean (SD) | 9.4 (9.2) | 8.6 (8.6) | 0.308 |
| Male, n (%) | 198 (57.4) | 86 (53.4) | 0.457 |
| Bulbar onset, n (%) | 93 (27.0) | 57 (35.4) | 0.067 |
| C9orf72 positive, n (%) | 13 (5.2) | 5 (3.5) | 0.617 |
| Behavioural involvement (Strong), n (%) | 120 (34.8) | 61 (37.9) | 0.562 |

*Complete-case patients had concurrent ECAS-behavioural, FrSBe and BBI assessment; excluded patients lacked one or more of these. Continuous variables compared by Mann-Whitney U test, categorical variables by chi-square or Fisher’s exact test. Behavioural involvement was defined as ALSbi, ALScbi or ALS-FTD on the Strong classification. SD, standard deviation.*

**Table S2. Item-level properties of the Frontal Behavioural Inventory (n = 294).**

| **Item** | **Description** | **Cluster** | **Item-total r** | **a** | **b** | **Endorsement** |
| --- | --- | --- | --- | --- | --- | --- |
| 1 | Apathy | Negative | 0.49 | 1.25 | +0.09 | 0.48 |
| 2 | Aspontaneity | Negative | 0.58 | 1.97 | +0.41 | 0.38 |
| 3 | Indifference | Negative | 0.64 | 2.48 | +0.87 | 0.24 |
| 4 | Inflexibility | Negative | 0.64 | 2.02 | +0.36 | 0.39 |
| 5 | Personal neglect | Negative | 0.59 | 1.66 | +1.24 | 0.19 |
| 6 | Disorganization | Negative | 0.70 | 2.60 | +0.62 | 0.30 |
| 7 | Inattention | Negative | 0.58 | 1.85 | +0.59 | 0.33 |
| 8 | Loss of insight | Negative | 0.52 | 1.86 | +0.50 | 0.36 |
| 9 | Logopenia | Negative | 0.57 | 1.39 | +0.09 | 0.48 |
| 10 | Semantic dementia | Negative | 0.48 | 1.32 | +1.78 | 0.14 |
| 11 | Aphasia/verbal apraxia | Negative | 0.28 | 0.68 | +1.23 | 0.32 |
| 12 | Alien hand/apraxia | Negative | 0.28 | 0.42 | +0.86 | 0.41 |
| 13 | Perseveration | Disinhibition | 0.54 | 1.56 | +1.11 | 0.23 |
| 14 | Irritability | Disinhibition | 0.50 | 0.99 | -0.10 | 0.52 |
| 15 | Jocularity | Disinhibition | 0.51 | 1.88 | +1.59 | 0.12 |
| 16 | Impulsivity | Disinhibition | 0.56 | 1.61 | +1.44 | 0.16 |
| 17 | Hoarding | Disinhibition | 0.42 | 2.30 | +2.21 | 0.04 |
| 18 | Inappropriateness | Disinhibition | 0.46 | 1.66 | +1.94 | 0.09 |
| 19 | Restlessness | Disinhibition | 0.41 | 1.17 | +1.89 | 0.14 |
| 20 | Aggression | Disinhibition | 0.42 | 1.00 | +1.94 | 0.16 |
| 21 | Hyperorality | Disinhibition | 0.37 | 1.15 | +2.50 | 0.09 |
| 22 | Hypersexuality | Disinhibition | 0.37 | 2.66 | +2.69 | 0.01 |
| 23 | Utilization | Disinhibition | 0.36 | 1.51 | +2.72 | 0.04 |
| 24 | Incontinence | Disinhibition | 0.32 | 0.83 | +2.46 | 0.14 |

*Item-total r, corrected item-total correlation. a and b, discrimination and difficulty parameters from a two-parameter item-response model fitted to dichotomised (endorsed vs not) item responses. Endorsement, proportion of patients endorsing the item (score ≥ 1). Cronbach’s α = 0.89 for the full scale (0.85 negative cluster, 0.79 disinhibition cluster); Kaiser-Meyer-Olkin = 0.86; Bartlett’s test p < 0.001.*

**Table S3. Convergent and divergent validity of the Frontal Behavioural Inventory.**

| **FBI measure vs comparator** | **Validity** | **n** | **Spearman ρ (95% CI)** | **p** |
| --- | --- | --- | --- | --- |
| FBI total vs BBI total | Convergent | 345 | +0.67 (+0.60 to +0.74) | <0.001 |
| FBI total vs FrSBe total | Convergent | 345 | +0.56 (+0.48 to +0.64) | <0.001 |
| FBI apathy vs FrSBe apathy | Convergent | 345 | +0.55 (+0.47 to +0.62) | <0.001 |
| FBI disinhibition vs FrSBe disinhibition | Convergent | 345 | +0.52 (+0.43 to +0.59) | <0.001 |
| FBI total vs HADS anxiety | Divergent | 292 | +0.10 (-0.02 to +0.21) | 0.098 |
| FBI total vs HADS depression | Divergent | 292 | +0.11 (-0.00 to +0.22) | 0.060 |

*Spearman correlations with 2000-resample bootstrap 95% confidence intervals. Convergent validity was tested against other behavioural instruments (BBI, FrSBe); divergent validity against the anxiety and depression subscales of the HADS. BBI, Beaumont Behavioural Inventory; FrSBe, Frontal Systems Behaviour Scale; HADS, Hospital Anxiety and Depression Scale.*

**Table S4. Sensitivity analysis — bulbar vs spinal onset.**

| **Subgroup** | **n** | **Gold prevalence** | **AUC** | **Youden cut-off (95% CI)** | **Sens / Spec / κ at ≥9** | **Sens / Spec / κ at ≥25** | **FBI mean ± SD (median)** |
| --- | --- | --- | --- | --- | --- | --- | --- |
| Bulbar | 93 | 29.0% | 0.875 | ≥ 15 (7–15) | 0.89 / 0.70 / +0.494 | 0.30 / 0.98 / +0.350 | 10.89 ± 11.31 (7) |
| Spinal | 252 | 23.0% | 0.848 | ≥ 9 (8–15) | 0.86 / 0.75 / +0.495 | 0.19 / 0.97 / +0.220 | 8.82 ± 8.28 (6) |

*Sensitivity analysis: FBI performance stratified by site of onset (bulbar vs spinal).*

**Table S5. Sensitivity analysis by Motor Severity (ALSFRS-R)**

*ROC recalibration of the FBI total cut-off stratified by ALSFRS-R severity (mild: ALSFRS-R > 35; moderate-severe: ALSFRS-R ≤ 35). AUC and Youden cut-off 95 % CIs are bootstrap-based (2 000 iterations). κ = Cohen’s kappa.*

| **Subgroup** | **n** | **Gold prev.** | **AUC** | **AUC 95% CI** | **Youden cut** | **Cut 95% CI** | **Sens @9** | **Spec @9** | **κ @9** | **Sens @25** | **Spec @25** |
| --- | --- | --- | --- | --- | --- | --- | --- | --- | --- | --- | --- |
| Mild (ALSFRS-R >35) | 206 | 24.6% | 0.8663 | 0.8033–0.9217 | 9 | 7–15 | 0.820 | 0.795 | +0.532 | 0.200 | 0.981 |
| Moderate-severe (ALSFRS-R <=35) | 66 | 37.9% | 0.8385 | 0.7346–0.9259 | 9 | 8–18 | 0.920 | 0.659 | +0.529 | 0.280 | 0.976 |

*Abbreviations: AUC, area under the ROC curve; CI, confidence interval; Sens, sensitivity; Spec, specificity; κ, Cohen’s kappa; @9 = FBI ≥ 9; @25 = FBI ≥ 25.*

**Table S6. Spearman correlations between FBI scores and ALSFRS-R domain scores.**

| **ALSFRS-R domain** | **n** | **FBI total rho (95% CI)** | **FBI total p** | **FBI Apathy rho (95% CI)** | **FBI Apathy p** | **FBI Disinhibition rho (95% CI)** | **FBI Disinhibition p** |
| --- | --- | --- | --- | --- | --- | --- | --- |
| Bulbar (items 1–3) | 247 | -0.204 [-0.33, -0.08] | 0.001 | -0.212 [-0.33, -0.09] | 0.001 | -0.118 [-0.25, 0.01] | 0.065 |
| Fine motor (items 4–6) | 247 | -0.167 [-0.29, -0.03] | 0.008 | -0.161 [-0.29, -0.03] | 0.052 | -0.124 [-0.25, 0.01] | 0.011 |
| Gross motor (items 7–9) | 247 | -0.101 [-0.22, 0.01] | 0.114 | -0.094 [-0.22, 0.02] | 0.143 | -0.085 [-0.20, 0.03] | 0.181 |
| Respiratory (items 10–12) | 246 | -0.115 [-0.24, 0.00] | 0.073 | -0.124 [-0.24, 0.00] | 0.053 | -0.045 [-0.17, 0.09] | 0.484 |
| Total | 272 | -0.203 [-0.315, -0.088] | 0.001 | -0.197 [-0.304, -0.082] | 0.001 | -0.154 [-0.271, -0.034] | 0.011 |

*Spearman rho with bootstrap 95% CI (1000 stratified resamples). ALSFRS-R higher = better function; negative rho indicates worse motor function associated with higher FBI scores. The threshold |rho| < 0.30 is the conventional criterion for absence of substantial method overlap (Streiner & Norman 2008; Gosselt et al. 2020). * flags |rho| >= 0.30.*

**Table S7. Distribution of FBI scores across decision bands.**

| **FBI score band** | **n** | **Impaired, n (%)** | **Wilson 95% CI** |
| --- | --- | --- | --- |
| FBI < 9 | 203 | 11 (5.4%) | 3.1%–9.4% |
| FBI 9-11 | 36 | 12 (33.3%) | 20.2%–49.7% |
| FBI 9-14 (grey zone) | 61 | 18 (29.5%) | 19.6%–41.9% |
| FBI 12-14 | 25 | 6 (24.0%) | 11.5%–43.4% |
| FBI >= 15 | 81 | 56 (69.1%) | 58.4%–78.1% |

*Complete-case subset (n = 345). Consensus-impaired = flagged by ≥2 of 3 FBI-independent instruments (ECAS-behavioural, FrSBe, BBI). Wilson (1927) score 95% confidence intervals are used instead of the normal-approximation interval because some bands contain few patients.*

**Table S8. Net benefit at representative threshold probabilities (Decision Curve Analysis).**

| **Strategy** | **pt = 0.10** | **pt = 0.20** | **pt = 0.25** | **pt = 0.30** | **pt = 0.40** | **pt = 0.50** |
| --- | --- | --- | --- | --- | --- | --- |
| Continuous FBI* | +0.1875 | +0.1544 | +0.1372 | +0.1249 | +0.1137 | +0.0756 |
| FBI >= 9 | +0.1928 | +0.1653 | +0.1487 | +0.1298 | +0.0823 | +0.0180 |
| FBI >= 15 | +0.154 | +0.144 | +0.138 | +0.131 | +0.114 | +0.090 |
| FBI >= 25 | +0.0532 | +0.0507 | +0.0493 | +0.0476 | +0.0434 | +0.0377 |
| Treat all | +0.1636 | +0.0583 | -0.0050 | -0.0775 | -0.2589 | -0.5048 |

*Net benefit NB(pt) = TP/N − (FP/N) × pt/(1−pt). Higher NB = greater clinical utility. Treat-none NB is always 0 (not shown). * Continuous FBI NB is optimistic: the logistic model is estimated and evaluated on the same sample (n = 345), whereas the fixed cut-off strategies have no free parameters and are thus unaffected by in-sample overfitting.*

**Table S9. FBI total score and diagnostic performance against the independent expert clinical (Strong ALS-FTSD) classification.**

*(a) FBI total score by Strong ALS-FTSD class*

| **Strong ALS-FTSD class** | **n** | **FBI median (IQR)** | **FBI mean (SD)** |
| --- | --- | --- | --- |
| ALS cognitively normal | 151 | 3 (1–6) | 5.2 (6.3) |
| ALS with cognitive impairment (ALSci) | 74 | 6 (3–10) | 8.5 (9.9) |
| ALS with behavioural impairment (ALSbi) | 52 | 12 (7–18) | 13.3 (8.4) |
| ALS with cognitive-behavioural impairment (ALScbi) | 55 | 15 (10–21) | 16.5 (8.6) |
| ALS-FTD | 13 | 17 (10–19) | 17.4 (10.9) |

*(b) FBI performance against the Strong-based reference standard (behavioural involvement: ALSbi, ALScbi or ALS-FTD)*

| **Cut-off** | **Sensitivity** | **Specificity** | **PPV** | **NPV** | **κ** |
| --- | --- | --- | --- | --- | --- |
| FBI ≥ 9 | 0.75 | 0.77 | 0.63 | 0.85 | +0.498 |
| FBI ≥ 15 | 0.51 | 0.91 | 0.75 | 0.78 | +0.454 |
| FBI ≥ 25 | 0.13 | 0.96 | 0.64 | 0.68 | +0.114 |

*The Strong classification was assigned by the treating clinicians at the time of assessment, independently of the caregiver behavioural questionnaires. Behavioural involvement was defined as ALSbi, ALScbi or ALS-FTD. Against this standard the FBI total achieved an AUC of 0.83; the Youden-optimal cut-off was 10 (bootstrap 95% CI 6 to 12). IQR, interquartile range; PPV, positive predictive value; NPV, negative predictive value; κ, Cohen’s kappa.*

**Figure S1.** Decision Curve Analysis of FBI cut-offs in ALS (n = 345). Net benefit is plotted against the threshold probability of behavioural impairment (p_t) for five strategies: FBI continuous (logistic model), FBI ≥ 9 (proposed screening cut-off), FBI ≥ 15 (exploratory higher-specificity cut-off), FBI ≥ 25 (legacy cut-off), and the treat-all and treat-none baselines. The shaded band (p_t 0.10–0.40) marks the clinically relevant range for behavioural screening in ALS. The vertical line marks the empirical prevalence (24.6%). Across the entire clinical range, FBI ≥ 9 and the continuous FBI model dominate the treat-all, treat-none and legacy ≥ 25 strategies; FBI ≥ 9 is preferable in the screening-oriented sub-range (p_t ≲ 0.30), while FBI ≥ 15 gives greater net benefit at higher threshold probabilities, supporting the exploratory two-threshold structure.

**Figure S2.** Item difficulty and discrimination of the Frontal Behavioural Inventory from a two-parameter item-response model (n = 294). Items are ordered by difficulty (b): negative/apathy items (blue) are generally endorsed at lower thresholds than disinhibition items (orange), consistent with the milder, apathy-predominant behavioural phenotype of ALS. Marker size is proportional to discrimination (a). The two motor or bulbar loaded items, aphasia/verbal apraxia and alien-hand/apraxia (red outline), show the lowest discrimination.
